# Circulating tRNA-derived small RNAs (tsRNAs) signature for the diagnosis and prognosis of breast cancer

**DOI:** 10.1038/s41523-020-00211-7

**Published:** 2021-01-05

**Authors:** Jingyi Wang, Ge Ma, Han Ge, Xu Han, Xinrui Mao, Xinyang Wang, Jordee Selvamanee Veeramootoo, Tiansong Xia, Xiaoan Liu, Shui Wang

**Affiliations:** 1 Department of Breast Surgery, The First Affiliated Hospital with Nanjing Medical University, 300 Guangzhou Road, 210029 Nanjing, China; 2 Jiangsu Key Lab of Cancer Biomarkers, Prevention and Treatment, Jiangsu Collaborative Innovation Center for Cancer Personalized Medicine, School of Public Health, Nanjing Medical University, 211166 Nanjing, China

**Keywords:** Diagnostic markers, Breast cancer

## Abstract

Liquid biopsy is noninvasive and convenient to detect cancer-derived materials in blood or other body fluids. The aim of this study was to identify tRNA-derived small RNAs (tsRNAs) in plasma that could distinguish patients with breast cancer (BC) from healthy controls. Basing on high-throughput sequencing, 15 significantly upregulated tsRNAs were selected and assessed in cell supernatants and cell lines. 6 tsRNAs were identified and verified in a large cohort of 120 patients with BC and 112 healthy controls. tRF-Arg-CCT-017, tRF-Gly-CCC-001, and tiRNA-Phe-GAA-003 could serve as novel diagnostic biomarkers. Meanwhile, tRF-Arg-CCT-017 and tiRNA-Phe-GAA-003 could also act as prognostic biomarkers. Target genes of these tsRNAs were related to the development of cancers. These results suggested that specific tsRNAs in plasma might serve as diagnostic and prognostic biomarkers of BC.

## Introduction

Breast cancer (BC) is the most common malignancy in women^1^. Liquid biopsy detects tumor-related materials (e.g., nucleic acid) in blood or other body fluids, and is helpful for diagnosis. Current researches on nucleic acid biomarkers are mainly about mircoRNAs, while few studies focus on tRNA-derived small RNAs (tsRNAs). tsRNAs, generated from precursor or mature tRNAs, are small non-coding RNAs, including tRNA-derived fragments (tRFs) and tRNA halves (tiRNAs)^2^. The classes of tRFs include tRF-1, tRF-2, tRF-3, and tRF-5, and tiRNAs are further classified into 5′tiRNA and 3′tiRNA^3^. Some studies have demonstrated that tsRNAs can be detected in blood and have potential to be diagnostic biomarkers of several diseases, including cancers^4–7^. In this study, high-throughput sequencing was used to identify differentially expressed tsRNAs in plasma samples between patients with BC and healthy controls. Cell supernatants and cell lines were used to screen tsRNAs, and 6 tsRNAs were selected to verify in a large cohort of plasma samples and exosomes. The diagnostic values of these tsRNAs were assessed, and disease-free survival rate (DFS) and overall survival rate (OS) were also compared. Furthermore, target genes, gene ontology, and pathways were analyzed. These results suggested that some specific tsRNAs in plasma could act as diagnostic and prognostic biomarkers of BC.

## Results

### Identification of differentially expressed tsRNAs in plasma

**Fig. 1:**
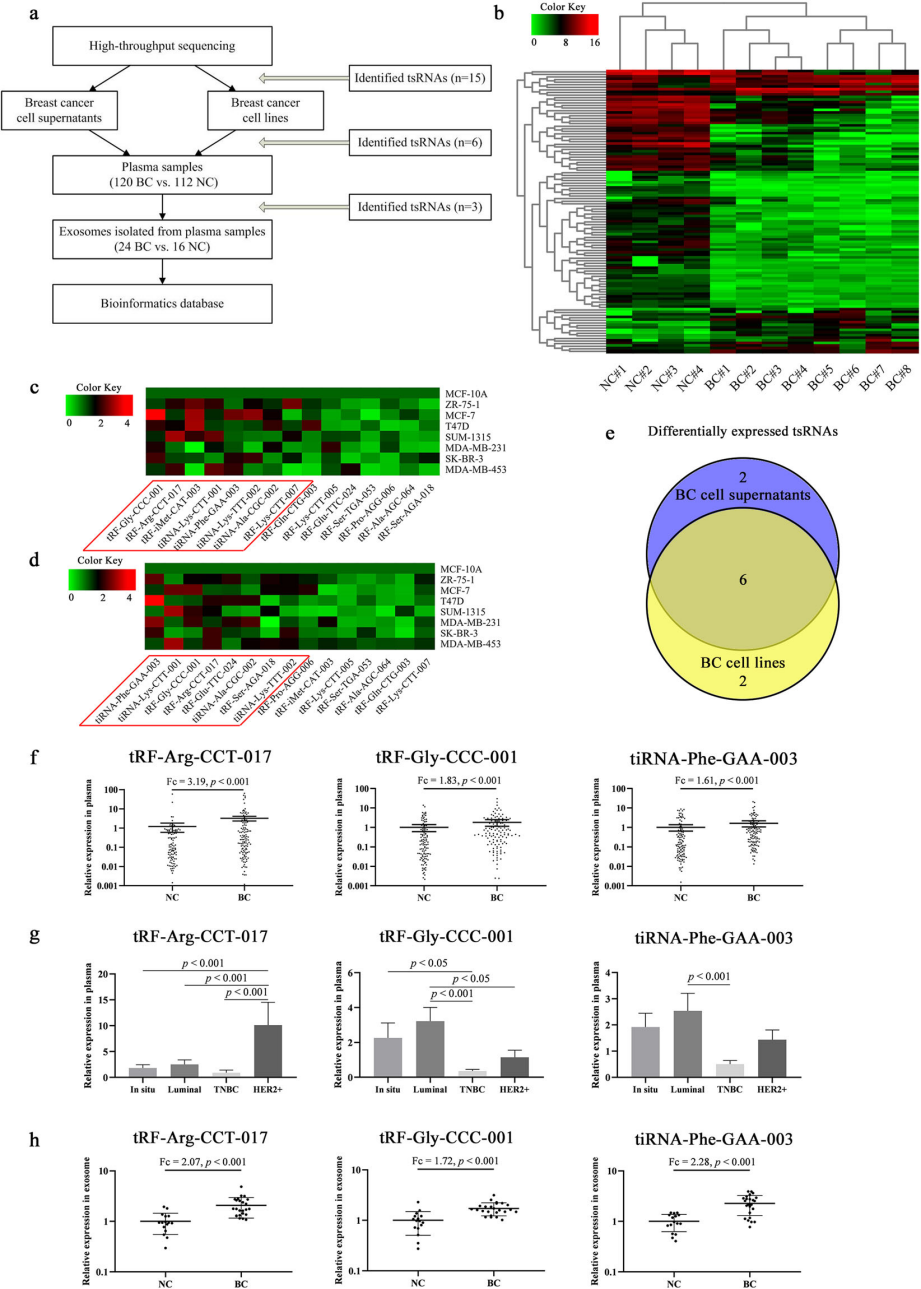
Identification of differentially expressed tsRNAs in plasma. **a** Flowchart of study design. **b** Heatmap clustering analysis of differentially expressed tsRNAs between 8 patients with BC and 4 healthy controls by high-throughput sequencing. **c** Relative expression levels of 15 tsRNAs in cell supernatants by qRT-PCR. The red box represented the expression levels in at least 3 BC cell supernatants were higher than that in human breast epithelial cell MCF-10A (fold change >1.5, *p* < 0.05). The color key meant fold change. **d** Relative expression levels in cell lines by qRT-PCR. The red box represented the expression levels in at least 3 BC cell lines were higher than that in MCF-10A (fold change >1.5, *p* < 0.05). The color key meant fold change. **e** Comparison of the results in cell supernatants and cell lines, and 6 tsRNAs were identified. **f** Expression levels of tRF-Arg-CCT-017, tRF-Gly-CCC-001, and tiRNA-Phe-GAA-003 in plasma samples from 120 patients with BC and 112 healthy controls by qRT-PCR. **g** Expression levels in plasma samples of different subtypes. **h** Expression levels in exosomes isolated from plasma samples of 24 patients with BC and 16 healthy controls by qRT-PCR. Horizontal line means with SEM. *Y* axis represents the relative expression (2^−ΔΔ*Ct*^). NC healthy controls, BC patients with breast cancer.

The study design was shown in Fig. 1a. High-throughput sequencing was used to compare the expression profile of plasma tsRNAs between 8 patients with BC and 4 healthy controls, and clustering analyses were performed (Fig. 1b). All differentially expressed tsRNAs were shown in Supplementary Table 1. Fifteen significantly upregulated tsRNAs in patients with BC were selected, and assessed in cell supernatants (Fig. 1c) and cell lines (Fig. 1d) by qRT-PCR. In at least 3 BC cell supernatants and at least 3 BC cell lines, the expression levels of tiRNA-Ala-CGC-002, tRF-Arg-CCT-017, tRF-Gly-CCC-001, tiRNA-Lys-CTT-001, tiRNA-Lys-TTT-002, and tiRNA-Phe-GAA-003 were higher than those in human breast epithelial cell line MCF-10A (fold change >1.5, *p* < 0.05) (Fig. 1e). Details of these tsRNAs were shown in Supplementary Table 2. These tsRNAs were verified in a large cohort of 120 patients with BC and 112 healthy controls by qRT-PCR, and the expression levels of tRF-Arg-CCT-017, tRF-Gly-CCC-001, and tiRNA-Phe-GAA-003 had statistical significances (*p* < 0.001) (Fig. 1f). The differences between four subtypes were also compared (Fig. 1g). The expression level of tRF-Arg-CCT-017 in HER-2 subtype was significantly higher than that in other subtypes, and the expression levels of tRF-Gly-CCC-001 and tiRNA-Phe-GAA-003 were different between Luminal and triple-negative breast cancer (TNBC), reflecting obvious heterogeneity. The expression levels of tRF-Arg-CCT-017, tRF-Gly-CCC-001, and tiRNA-Phe-GAA-003 were also assayed in exosomes isolated from plasma samples of 24 patients with BC and 16 healthy controls by qRT-PCR, and all showed significant differences (*p* < 0.001) (Fig. 1h). Exosomes were verified by Western blot and nanoparticle tracking analysis (Supplementary Fig. 1).

### The diagnostic and prognostic values of identified tsRNAs

**Fig. 2:**
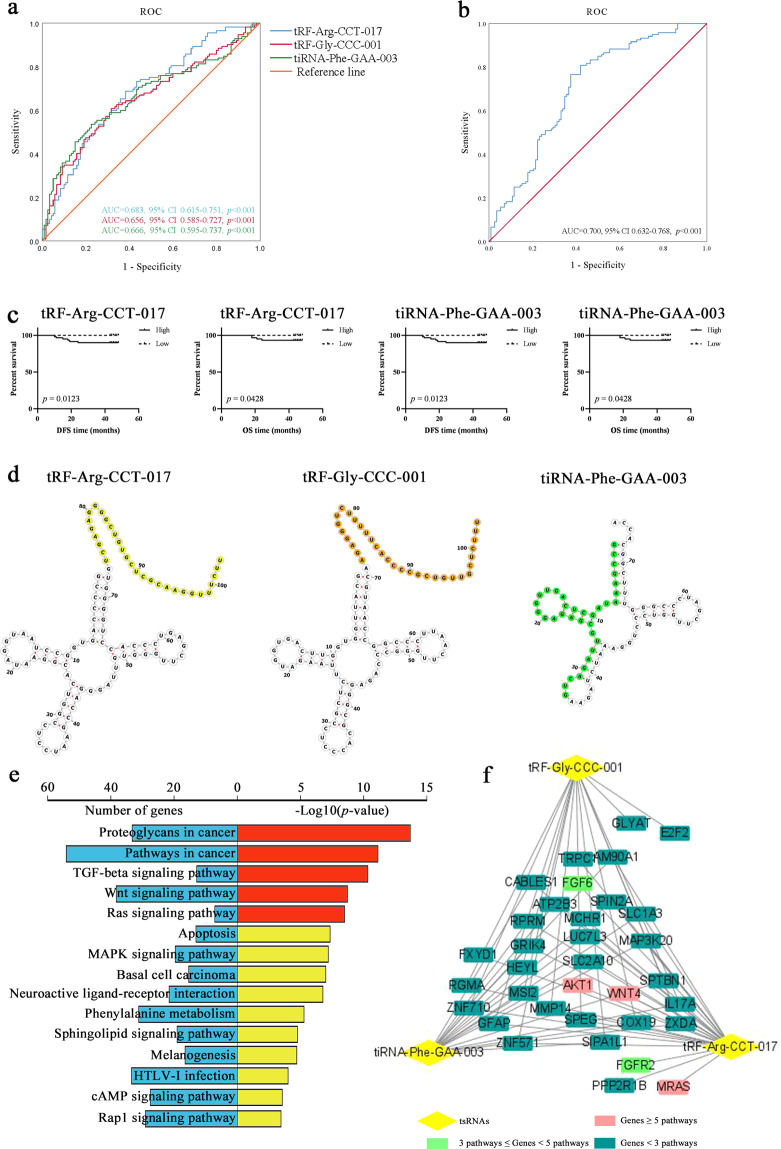
The diagnostic and prognostic values of identified tsRNAs. **a** ROC curves of three tsRNAs for distinguishing patients with BC from healthy controls. **b** ROC curves of the three-tsRNAs panel. **c** High expression levels of tRF-Arg-CCT-017 and tiRNA-Phe-GAA-003 indicated worse DFS and OS. **d** Diagrams of secondary structures predicted by Vienna RNA Web Services. tRF-Arg-CCT-017 and tRF-Gly-CCC-001 were tRF-1 fragments, and tiRNA-Phe-GAA-003 was a 5′tiRNA fragment. **e** KEGG pathways enriched for tRF-Arg-CCT-017, tRF-Gly-CCC-001, and tiRNA-Phe-GAA-003, and most were cancer-related. **f** tsRNAs-gene connection network. Yellow rhombuses represent tsRNAs. The pink, green, and blue rectangles represent target genes that were involved in ≥5, 3–5, and <3 significant pathways, respectively.

Receiver operating characteristic (ROC) curves were used to evaluate diagnostic values. The area under the curves (AUCs) of tRF-Arg-CCT-017, tRF-Gly-CCC-001, and tiRNA-Phe-GAA-003 were 0.683 (95%CI 0.615–0.751, *p* < 0.001), 0.656 (95%CI 0.585–0.727, *p* < 0.001) and 0.666 (95%CI 0.595–0.737, *p* < 0.001) (Fig. 2a), suggesting the specificity of diagnosis. The AUC for the three-tsRNAs panel was 0.700 (95%CI 0.632–0.768, *p* < 0.001) (Fig. 2b). The combination improved accuracy, and a logistic regression model was performed for prediction. The predictive probability was calculated using the formula as follows: Logit(*P*) = 0.288 − 0.126 × tRF-Arg-CCT-017 − 0.022 × tRF-Gly-CCC-001 − 0.123 × tiRNA-Phe-GAA-003. According to expression levels, patients were divided into high-expression group or low-expression group. We found that patients with high levels of tRF-Arg-CCT-017 or tiRNA-Phe-GAA-003 were related to worse DFS and OS (Fig. 2c). There was no difference of tRF-Gly-CCC-001 (Supplementary Fig. 2).

### Bioinformatics analysis of identified tsRNAs

Diagrams of secondary structures were predicted by Vienna RNA Web Services (Fig. 2d). tRF-Arg-CCT-017 and tRF-Gly-CCC-001, generated from precursor tRNA, were tRF-1 fragments, and tiRNA-Phe-GAA-003, derived from mature tRNA, was a 5′tiRNA fragment. To gain further insight of these tsRNAs, KEGG pathways of target genes were performed. Fifteen significant pathways were involved (*p* < 0.05) and most were cancer-related, such as TGF-beta signaling pathway, Wnt signaling pathway (Fig. 2e). The connection network showed targeted genes. These tsRNAs regulated genes AKT1, MRAS, and WNT4, which were involved in more than 5 of the 15 pathways, and also regulated other important genes, including FGF6, FGFR2, and MMP14 (Fig. 2f).

## Discussion

The 5-year survival rate of early-stage breast cancer is obviously better than that of advanced stage^8^. Liquid biopsy is noninvasive and helpful for early diagnosis. tsRNAs, a class of non-coding small RNAs, can inhibit protein translation and regulate gene expression among other less studied functions^9–11^. Our study focused on differentially expressed tsRNAs in plasma between patients with BC and healthy controls, and we found the diagnostic values of tRF-Arg-CCT-017, tRF-Gly-CCC-001, and tiRNA-Phe-GAA-003, and the prognostic values of tRF-Arg-CCT-017 and tiRNA-Phe-GAA-003. The accuracy of the three-tsRNAs panel was better than any tsRNAs. Except plasma, the expression levels in exosomes were also analyzed, and the tendencies were consistent with those in plasma. There was no correlation of tRF-Arg-CCT-017, while there were obvious correlations of tRF-Gly-CCC-001 and tiRNA-Phe-GAA-003, indicating that most tRF-Gly-CCC-001 and tiRNA-Phe-GAA-003 in plasma were existed in exosomes (Supplementary Fig. 3). RNAs in exosomes are relatively more stable and resistant to physical degradation, but it is more convenient to detect RNAs in plasma than those in exosomes. We inferred these tsRNAs may be related to the progression of BC. tRF-Gly-CCC-001 has 5 Ts in a row, while 4 Ts is a transcriptional stop sequence for RNA Pol III. The sequence analysis of tRF-Gly-CCC-001 showed a high degree of similarity with Human Gly-tRNA gene using the basic local alignment search tool (BLAST) function of the National Center for Biotechnology Information (NCBI) (https://blast.ncbi.nlm.nih.gov/Blast.cgi), suggesting that this tsRNA could continue with 5Ts. 5 Ts in a row may be relatively unstable, while the development of BC may promote its stability. Moreover, target genes were associated with the development of cancers. Functional investigation may give insights into their mechanisms. In conclusion, the identified tsRNAs in plasma are valuable diagnostic and prognostic biomarkers of BC.

## Methods

### Samples collection

A total of 128 patients with BC without neoadjuvant therapy and 116 healthy controls were recruited from the First Affiliated Hospital with Nanjing Medical University from January to December 2015, with a mean follow-up from diagnosis of 4 years. Clinical characteristics were provided in Supplementary Table 3. Peripheral venous blood samples (2 mL) were collected and centrifuged at 3000 *g* for 10 min at 4 °C to harvest plasma within 4 h before storing at −80 °C. The methods were performed in accordance with relevant guidelines and regulations and approved by the institutional ethical committee of the First Affiliated Hospital with Nanjing Medical University. Patients recruited in this study provided informed written consent.

### High-throughput sequencing

Before cDNA library construction, modifications in tsRNAs were removed by rtStar™ tRF&tiRNA Pretreatment Kit (Arraystar, USA), which removes 3′-aminoacyl and 3′-cyclic phosphate for 3′-adaptor ligation, phosphorylates 5′-OH for 5′-adaptor ligation, and demethylates m1A, m1G, and m3C. cDNA was then synthesized and amplified. Subsequently, ~134–160 bp PCR amplified fragments were extracted and purified from the PAGE gel. The completed libraries were quantified by Agilent 2100 Bioanalyzer (Agilent, USA), and then sequenced on Illumina NextSeq 500 system (Illumina, USA). After generating raw sequencing data, intronic sequences were removed and “CCA” were added to 3′-terminal to generate mature tRNA libraries. 40 nucleotides of flanking genomic sequence on either side of original tRNA sequence were included for precursor tRNA libraries^12^. The mature or precursor tRNA sequences were downloaded from GtRNAdb (http://gtrnadb.ucsc.edu/). Trimmed reads were aligned allowing for 1 mismatch only to the mature tRNA sequences, then reads that do not map were aligned allowing for 1 mismatch only to precursor tRNA sequences^13^. The abundance of each tsRNA was evaluated using sequencing count and was normalized as reads per million of total aligned reads (RPM). Our study did not involve in the content of ‘Guidance of the Ministry of Science and Technology (MOST) for the Review and Approval of Human Genetic Resources’.

### Cell culture and supernatants collection

All cell lines were obtained from ATCC (Manassas, USA). All cells were seeded with same density, and supernatants were collected after 24 h and centrifuged at 3000 *g* for 10 min at 4 °C before storing at −80 °C.

### Isolation of exosomes

Plasmas were first treated with thrombin and then mixed with Exo-Quick exosome precipitation solution according to the manufacturer’s protocol. After removing the supernatants, exosome pellets were lysed in RNase-free water for further RNA extraction.

### RNA extraction and qRT-PCR

Total RNA from cells was extracted by RNAiso plus (TaKaRa, Japan), and total RNA from supernatants, plasma, and exosomes were isolated by mirVana PARIS Kit (Invitrogen, Lithuania). The quality and quantity were determined by NanoDrop 1000 (ThermoFisher Scientific, MA). Each RNA was reverse transcribed to cDNA by the Bulge-loop™ qRT-PCR Primer Sets (Ribobio, China), specially for tsRNAs^14^. The bulge-loop reverse transcription primers were 40–60 nucleotides. From 5′ to 3′, the primer included stem 1, bulge, stem 2, ring, stem 3, and extension. Stem 3 was complementary with stem 1 and stem 2, and the extension was complementary with small RNA. The primers inhibit binding to sequences of full-length tRNAs. The PCR forward primers were 20–40 nucleotides, with extensions and sequences similar to small RNAs, and general reverse primers were 20–40 nucleotides. Standard RNAs, which were identical to target sequence and acted as positive controls, were used to verify the specificity of primers (Supplementary Fig. 4). The qRT-PCR was run on the Roche LightCycler^®^ 480 System (Roche, Switzerland). Relative expression levels were normalized to RNU6B (U6) and analyzed by the 2^−∆∆*Ct*^ method.

### Statistical analysis

Mann–Whitney test was used to compare expression levels. ROC analysis was performed to determine AUC and logistic regression by SPSS. For analysis of biomarkers combination, logistic regression model was applied and probability was calculated by the formula: Logit(*P*) = *b*_0_ + *b*_1_*∆*CT*_1_ + *b*_2_*∆*CT*_2_ + *b*_3_*∆*CT*_3_… + *b*_n_*∆*CT*_n_, where the *b*_*i*_ meant the *i*th regression coefficients by binary logistic regression, and the ∆*CT*_i_ meant the relative expression level of each biomarker^15,16^. In our study, the model was applied on the expression levels of the 3 tsRNAs in plasma, and was used to establish the three-tsRNAs panel. DFS and OS were analyzed by Kaplan–Meier curves. Sequences of tsRNAs were downloaded from GtRNAdb (http://gtrnadb.ucsc.edu/), and diagrams of secondary structures were drawn by Vienna RNA Web Services (http://rna.tbi.univie.ac.at/forna/). The significances of KEGG pathway enrichment were tested by two-sided Fisher’s exact test and Chi-squared test. Cytoscape was used to draw the network of target genes. All statistical analysis and graph plotting were performed by SPSS 25.0 and GraphPad Prism 8.0. *P* < 0.05 was defined as statistical significance.

### Reporting summary

Further information on research design is available in the Nature Research Reporting Summary linked to this article.

## Supplementary information

Supplementary files

Reporting Summary

## Data Availability

The data generated and analyzed during this study are described in the following metadata record: 10.6084/m9.figshare.12961883^17^. The expression data are available in the Gene Expression Omnibus repository under accession: https://identifiers.org/geo:GSE158945^18^.
