# Supplementary material for: Circulating tRNA-derived small RNAs (tsRNAs) signature for the diagnosis and prognosis of breast cancer
Source: NPJ Breast Cancer. 2021 Jan 5;7:4. doi: 10.1038/s41523-020-00211-7 (PMC7785726; doi:10.1038/s41523-020-00211-7)
Supplement: Supplementary file 1 — Supplementary files [file 41523_2020_211_MOESM1_ESM.pdf]

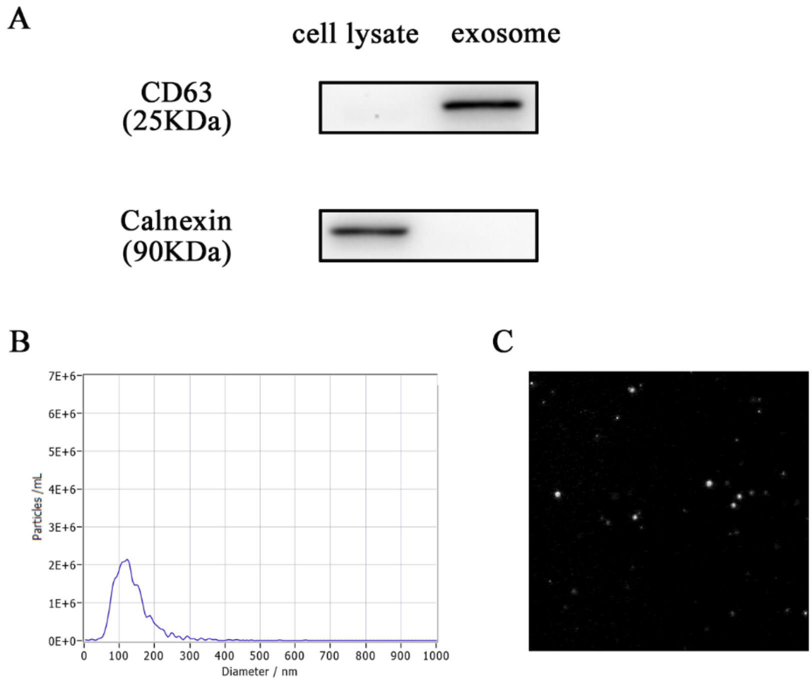

Supplementary Figure 1. The verification of exosomes isolated from plasma. **a** Western blot analysis showed the protein expression levels of exosome marker (CD63) and non-exosome marker (Calnexin) in cell lysate of MCF-10A and exosome. **b** Exosomes were verified by nanoparticle tracking analysis (NTA), and the diameters were mainly between 80 - 150 nm. **c** A screenshot of NTA.

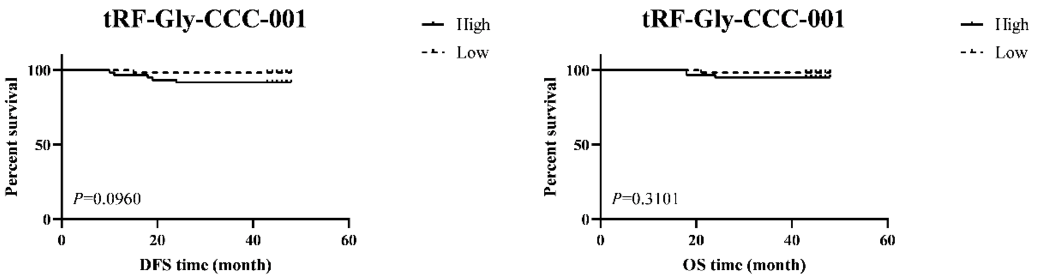

Supplementary Figure 2. No differences in DFS and OS between high-expression level and low-expression level of tRF-Gly-CCC-001.

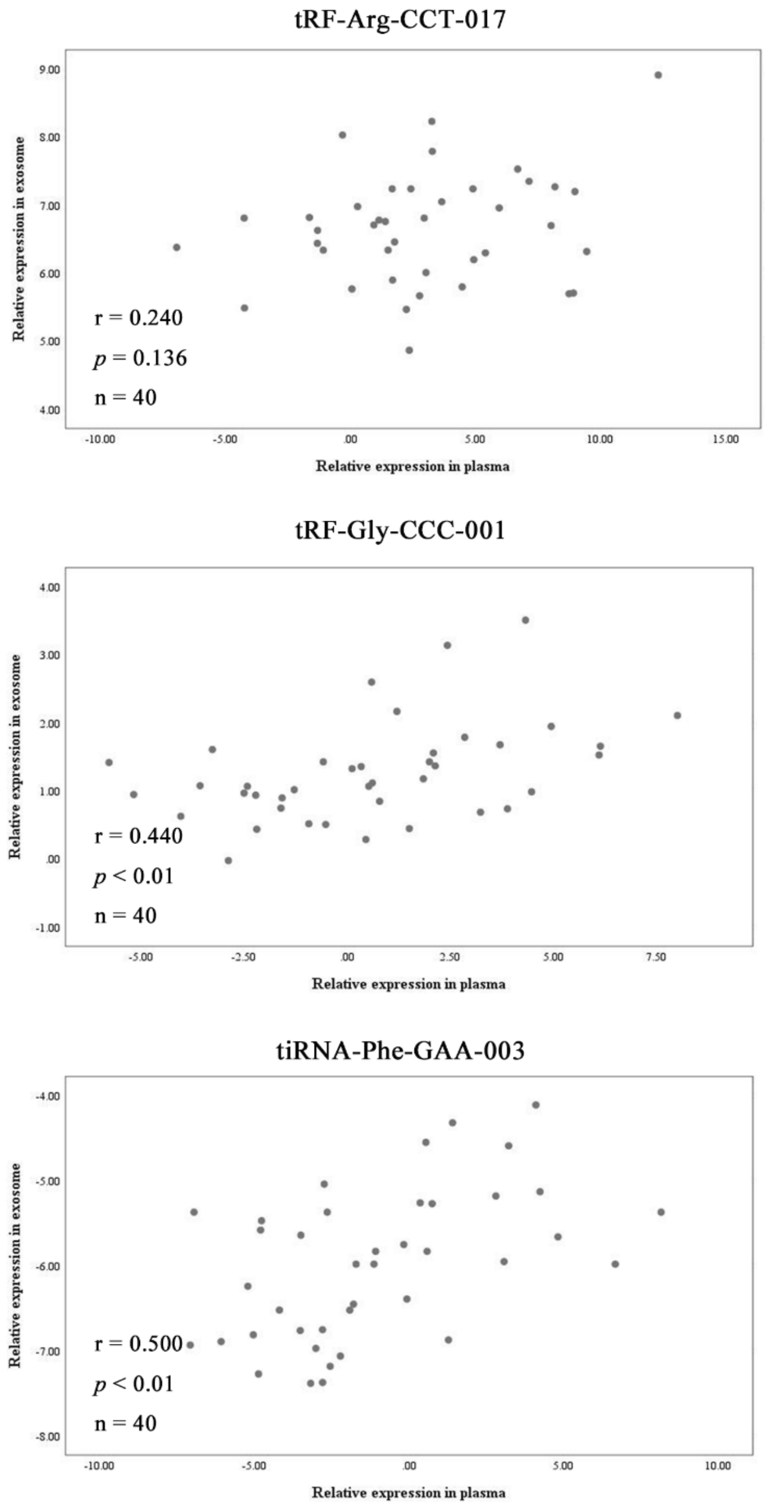

Supplementary Figure 3. The correlations between the expression levels in plasma samples and exosomes. Obvious correlations of tRF-Gly-CCC-001 and tiRNA-Phe-GAA-003, but no correlation of tRF-Arg-CCT-017.

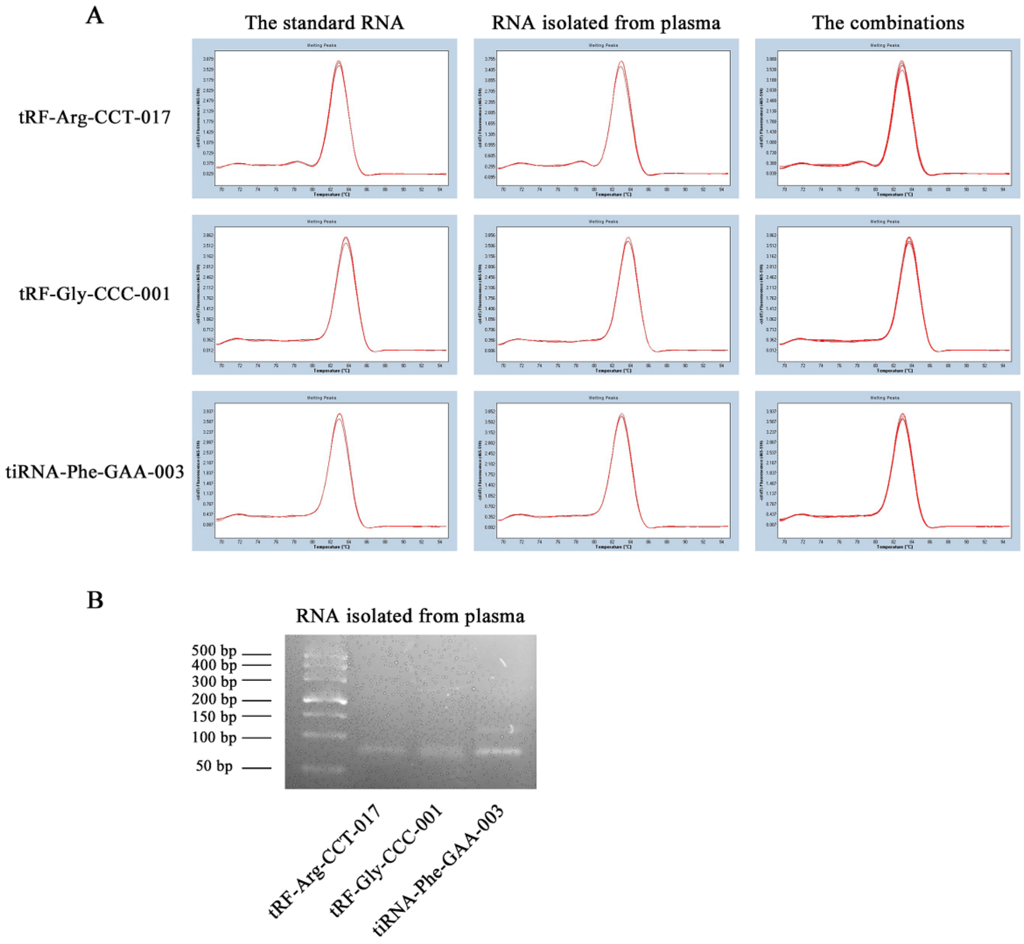

Supplementary Figure 4. The specificity of primers. **a** Melting curve and melting temperature of each primer in standard RNA, RNA isolated from plasma samples, and their combinations. **b** Analysis of qRT-PCR products of RNA isolated from plasma samples by agarose gel electrophoresis (AGE).

Supplementary Figure 1a

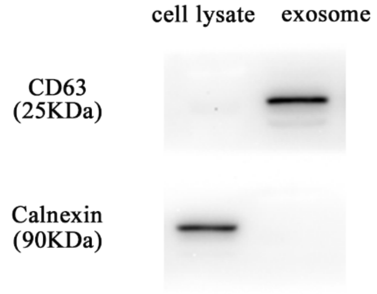

Supplementary Figure 4b

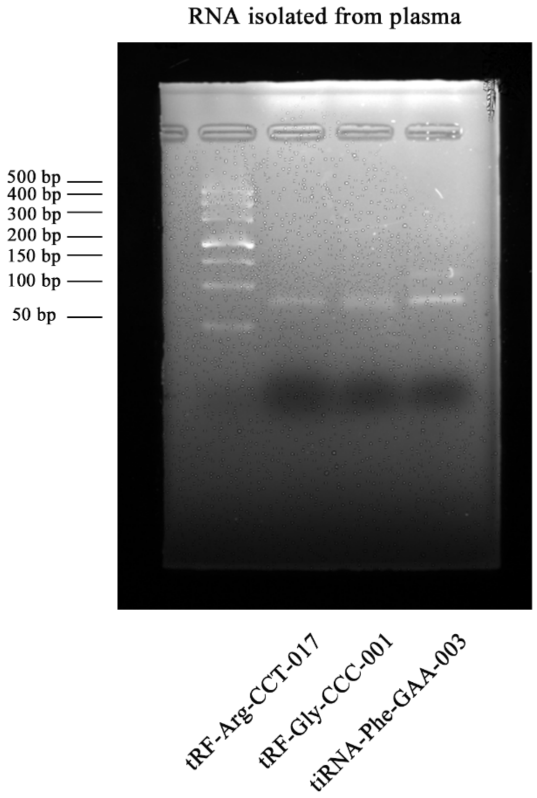

Supplementary Figure 5. Raw data for the blots. All the blots derived from the same experiment and were processed in parallel.

Supplementary Table 1. Differentially expressed tsRNAs between 8 patients with BC and 4 healthy controls by high-throughput sequencing. The bold were 15 significantly upregulated tsRNAs in patients with BC, and were assessed in cell supernatants and cell lines. NC, healthy controls; BC, patients with breast cancer.

tsRNAs	Type	Length	NC	BC	FoldChange	*p*-value
**tRF-Gly-CCC-001**	**tRF-1**	**32**	**0.000±0.000**	**195.043±406.667**	**561.204**	**0.000**
**tiRNA-Ala-CGC-002**	**tiRNA-5**	**33**	**0.000±0.000**	**34.276±96.948**	**101.525**	**0.011**
**tRF-Arg-CCT-017**	**tRF-1**	**30**	**0.000±0.000**	**43.552±68.549**	**91.857**	**0.015**
**tiRNA-Phe-GAA-003**	**tiRNA-5**	**34**	**0.000±0.000**	**25.276±71.491**	**74.704**	**0.025**
**tiRNA-Lys-CTT-001**	**tiRNA-5**	**34**	**3.235±6.471**	**81.992±161.07**	**27.688**	**0.017**
**tRF-Lys-CTT-005**	**tRF-5c**	**29**	**4.253±8.505**	**74.703±71.475**	**27.057**	**0.016**
**tRF-Ser-TGA-053**	**tRF-3b**	**22**	**8.255±12.038**	**105.123±196.628**	**22.411**	**0.011**
**tRF-iMet-CAT-003**	**tRF-5c**	**32**	**1.618±3.235**	**45.556±66.172**	**22.205**	**0.045**
**tRF-Pro-AGG-006**	**tRF-5c**	**32**	**137.876±39.635**	**1484.093±1420.891**	**17.096**	**0.002**
**tiRNA-Lys-TTT-002**	**tiRNA-5**	**34**	**34.835±20.048**	**244.485±151.901**	**12.871**	**0.014**
**tRF-Ala-AGC-064**	**tRF-3b**	**22**	**19.158±11.59**	**119.547±118.667**	**11.902**	**0.028**
**tRF-Ser-AGA-018**	**tRF-3b**	**22**	**46.944±68.72**	**255.68±531.274**	**10.019**	**0.025**
**tRF-Gln-CTG-003**	**tRF-5c**	**30**	**177.255±69.143**	**948.729±863.042**	**8.836**	**0.015**
**tRF-Lys-CTT-007**	**tRF-5c**	**31**	**38.325±26.363**	**189.915±220.094**	**8.243**	**0.041**
**tRF-Glu-TTC-024**	**tRF-5c**	**31**	**1200.701±395.837**	**6065.475±4860.315**	**8.058**	**0.010**
tRF-Pro-AGG-002	tRF-5c	28	56.462±22.495	260.128±167.663	7.888	0.038
tRF-Lys-CTT-004	tRF-5c	28	37.716±28.282	205.038±184.503	7.684	0.048
tRF-Gln-TTG-003	tRF-5c	30	96.651±46.023	457.701±439.87	7.300	0.034
tRF-Glu-TTC-021	tRF-5c	32	58.968±68.028	311.338±277.946	7.066	0.046
tRF-Val-TAC-012	tRF-5c	31	353.323±440.008	1600.428±1117.942	6.739	0.025
tRF-Arg-CCT-001	tRF-1	31	88.432±89.287	288.384±301.38	6.565	0.049
tRF-Val-AAC-015	tRF-5c	31	284.631±326.512	941.05±814.59	6.464	0.032
tRF-Pro-AGG-003	tRF-5c	29	900.363±988.588	3022.106±2562.979	6.177	0.027
tRF-His-GTG-006	tRF-5c	29	207.809±222.736	609.899±417.178	5.820	0.047
tRF-Lys-CTT-008	tRF-5c	32	576.122±460.514	1884.091±836.39	5.507	0.040
tRF-Gly-CCC-006	tRF-5a	16	4040.763±2347.859	18.996±21.531	0.007	0.000
tRF-Tyr-GTA-034	tRF-5c	28	89.198±72.596	0.000±0.000	0.008	0.000
tRF-Ala-AGC-047	tRF-5a	16	904.233±216.856	5.794±8.916	0.010	0.000
tRF-Gly-GCC-033	tRF-5b	23	95.089±190.178	0.000±0.000	0.010	0.001
tRF-Lys-TTT-028	tRF-5c	28	73.785±39.011	0.000±0.000	0.011	0.001
tRF-Glu-CTC-003	tRF-5a	16	120.854±128.688	0.265±0.75	0.012	0.001
tRF-Gly-CCC-007	tRF-5b	22	36.765±50.864	0.000±0.000	0.019	0.007
tRF-Ser-TGA-001	tRF-5b	24	1125.436±672.253	13.533±20.4	0.020	0.000
tRF-Gly-CCC-008	tRF-5b	23	81.762±29.055	0.836±2.365	0.022	0.003
tRF-Ser-TGA-014	tRF-3a	18	25.3±33.702	0.000±0.000	0.025	0.012
tRF-Gly-CCC-009	tRF-5b	24	30.589±31.112	0.000±0.000	0.027	0.013

tiRNA-Gln-TTG-001	tiRNA-5	34	25.988±20.041	0.000±0.000	0.027	0.013
tRF-Ser-TGA-002	tRF-5c	28	68.235±74.936	0.69±1.952	0.027	0.005
tRF-Leu-CAA-003	tRF-5a	16	1899.16±2332.96	28.146±46.262	0.028	0.000
tRF-Val-TAC-020	tRF-3b	22	23.849±16.643	0.000±0.000	0.028	0.015
tiRNA-Met-CAT-001	tiRNA-5	31	31.696±63.393	0.000±0.000	0.030	0.017
tRF-Gly-GCC-032	tRF-5a	16	20.096±14.016	0.000±0.000	0.032	0.020
tRF-Tyr-GTA-035	tRF-5c	29	20.504±31.865	0.000±0.000	0.033	0.020
tRF-Gly-CCC-015	tRF-5a	14	2115.079±812.219	49.29±42.23	0.034	0.000
tRF-Leu-AAG-008	tRF-3b	19	582.113±294.975	11.07±16.783	0.035	0.000
tRF-Val-TAC-006	tRF-5c	31	18.378±27.639	0.000±0.000	0.037	0.027
tiRNA-Thr-TGT-001	tiRNA-3	37	360.365±140.996	9.026±13.08	0.037	0.000
tRF-Glu-CTC-002	tRF-5a	15	178.804±205.415	3.742±5.907	0.038	0.001
tRF-Ala-AGC-041	tRF-5c	29	17.47±20.283	0.000±0.000	0.039	0.027
tRF-Ser-TGA-022	tRF-5a	16	150.553±214.064	3.232±5.744	0.039	0.002
tRF-Cys-GCA-015	tRF-5b	22	23.772±47.545	0.000±0.000	0.040	0.032
tRF-Gly-CCC-050	tRF-1	31	23.772±47.545	0.000±0.000	0.040	0.032
tRF-Val-CAC-029	tRF-5a	16	156.314±149.613	2.873±7.262	0.040	0.003
tRF-Gln-TTG-004	tRF-5a	15	253.646±161.434	6.195±7.612	0.041	0.001
tRF-Leu-TAA-009	tRF-2	15	21.477±29.892	0.000±0.000	0.042	0.032
tRF-Gly-CCC-035	tRF-5a	15	341.729±352.077	9.418±17.463	0.043	0.000
tRF-Ser-TGA-004	tRF-5c	30	82.283±33.664	1.577±3.639	0.045	0.011
tRF-Thr-CGT-015	tRF-1	19	14.884±29.768	0.000±0.000	0.045	0.039
tRF-Leu-TAG-013	tRF-5c	31	14.375±24.627	0.000±0.000	0.045	0.039
tRF-Ser-TGA-020	tRF-5a	14	942.395±663.978	27.263±35.915	0.046	0.000
tRF-Leu-TAG-015	tRF-3a	18	109.108±48.157	2.706±4.17	0.046	0.005
tRF-Gly-CCC-016	tRF-5a	16	99.97±96.458	2.451±3.641	0.046	0.007
tRF-Gly-CCC-017	tRF-5b	22	77.96±71.14	1.966±5.56	0.046	0.011
tRF-Cys-GCA-013	tRF-5a	15	28.457±24.053	0.281±0.794	0.048	0.015
tRF-Gly-CCC-036	tRF-5a	15	151.3±98.689	3.574±4.322	0.048	0.004
tRF-Gly-GCC-012	tRF-3b	22	1418.464±1002.976	47.612±69.657	0.048	0.000
tRF-Leu-TAA-006	tRF-3a	18	304.715±259.729	10.032±26.817	0.050	0.001
tiRNA-Lys-TTT-001	tiRNA-5	29	75.614±60.07	2.224±3.812	0.050	0.011
tRF-Ala-TGC-002	tRF-5a	14	14.752±25.374	0.000±0.000	0.052	0.047
tRF-Asn-GTT-038	tRF-2	16	26.576±18.436	0.265±0.75	0.053	0.017
tRF-Ser-TGA-021	tRF-5a	15	34.006±39.107	0.493±1.394	0.053	0.035
tRF-Gly-CCC-002	tRF-5a	14	119.287±88.147	4.205±9.313	0.056	0.004
tRF-Met-CAT-007	tRF-5a	14	3054.326±1091.8	107.196±99.787	0.059	0.000
tRF-Cys-GCA-011	tRF-3a	17	1991.722±1104.134	66.452±70.129	0.060	0.000
tRF-Gly-GCC-020	tRF-3b	20	49.083±48.58	1.479±4.182	0.061	0.027
tRF-Asn-GTT-037	tRF-2	14	88.291±73.137	2.645±4.05	0.061	0.015
tRF-Leu-TAA-036	tRF-5a	16	23.099±28.969	0.493±1.394	0.063	0.045
tiRNA-Ser-TGA-001	tiRNA-5	31	46.497±40.368	1.479±4.182	0.064	0.029
tRF-Gly-CCC-046	tRF-3a	18	286.556±135.348	11.111±24.469	0.065	0.002
tRF-Val-CAC-015	tRF-3a	18	2323.931±1423.217	93.837±114.992	0.066	0.000

tRF-Gly-CCC-041	tRF-3a	17	118.816±47.407	5.152±10.887	0.071	0.010
tRF-Gly-GCC-001	tRF-3b	21	45.33±42.201	1.634±3.11	0.073	0.031
tRF-Ser-TGA-024	tRF-5b	23	452.46±273.901	15.629±30.418	0.074	0.003
tRF-Leu-CAG-001	tRF-3b	19	191.172±69.343	8.254±13.238	0.075	0.007
tRF-Gly-GCC-037	tRF-3b	22	67.299±16.379	2.862±3.974	0.076	0.017
tRF-Cys-GCA-029	tRF-1	25	13.718±16.341	0.281±0.794	0.077	0.039
tRF-Val-CAC-007	tRF-5c	28	42.187±49.89	1.678±2.344	0.078	0.035
tRF-Ser-TGA-035	tRF-3b	22	14.485±13.054	0.281±0.794	0.079	0.039
tRF-Ser-TGA-017	tRF-3a	17	13.882±2.765	0.281±0.794	0.085	0.047
tRF-Pro-TGG-005	tRF-5c	29	91.505±131.381	4.417±7.714	0.102	0.040
tRF-Leu-CAG-002	tRF-3a	18	584.376±166.018	40.601±43.697	0.110	0.005
tRF-Gly-GCC-034	tRF-5b	24	316.859±125.591	24.957±35.02	0.116	0.010
tiRNA-Gly-TCC-001	tiRNA-5	34	1920.263±669.949	111.594±147.017	0.120	0.003
tRF-Trp-CCA-027	tRF-3b	22	96.76±63.866	7.029±13.144	0.125	0.044
tRF-His-GTG-012	tRF-3b	22	92.796±90.227	8.144±17.552	0.130	0.043
tRF-Gln-TTG-035	tRF-5a	14	348.349±323.28	30.185±45.396	0.132	0.013
tRF-Gln-TTG-005	tRF-5a	16	477.171±290.968	40.19±63.87	0.134	0.011
tRF-Ala-AGC-004	tRF-5a	16	408.322±87.447	36.567±38.219	0.141	0.015
tRF-Gly-CCC-045	tRF-3a	18	141.317±101.981	15.01±25.142	0.148	0.036
tRF-Pro-AGG-001	tRF-3a	18	6491.619±3340.314	677.238±1294.51	0.154	0.004
tRF-Gly-TCC-014	tRF-5c	30	1490.278±818.759	158.226±211.961	0.169	0.012
tRF-Gly-TCC-011	tRF-5b	23	273.769±75.734	29.305±33.108	0.178	0.038
tRF-Val-AAC-001	tRF-3a	18	7906.874±4742.272	1027.111±1235.171	0.203	0.014
tRF-Pro-AGG-025	tRF-3a	17	1166.227±547.475	160.694±273.768	0.205	0.026
tRF-Gly-CCC-043	tRF-3b	22	526.298±212.796	71.701±98.44	0.208	0.039
tRF-Ser-AGA-001	tRF-3a	18	929.34±537.315	120.132±144.953	0.214	0.032
tiRNA-Ser-GCT-001	tiRNA-5	20	1132.663±966.241	149.629±115.383	0.215	0.030
tRF-Cys-GCA-002	tRF-3a	18	652.479±346.256	94.492±176.796	0.217	0.040
tRF-Ser-TGA-003	tRF-5c	29	1430.643±649.399	163.076±251.62	0.229	0.036

Supplementary Table 2. Details of the selected tsRNAs.

tsRNAs	Type	Sequence	Length
tiRNA-Ala-CGC-002	tiRNA-5	GGGGATGTAGCTCAGTGGTAGAGCGCATGCTTC	33
tRF-Arg-CCT-017	tRF-1	TCGAGAGGGGCTGTGCTCGCAAGGTTTCTT	30
tRF-Gly-CCC-001	tRF-1	AGAGGGTCTTTTTCACCCCGCTGTTGCTCTTT	32
tiRNA-Lys-CTT-001	tiRNA-5	GCCCGGCTAGCTCAGTCGGTAGAGCATGAGACCC	34
tiRNA-Lys-TTT-002	tiRNA-5	GCCCGGATAGCTCAGTCGGTAGAGCATCAGACTT	34
tiRNA-Phe-GAA-003	tiRNA-5	GCCGAAATAGCTCAGTTGGGAGAGCGTTAGACTG	34

Supplementary Table 3. Clinical characteristics of all participants in the study. NC, healthy controls; BC, patients with breast cancer.

**Characteristics**	NC	BC
**Number**	116	128
**Age**		
< 50	60	60
≥ 50	56	68
**TNM stage**		
ductal carcinoma in situ		19
I		10
II		57
III		42
**Grade**		
I		10
II		59
III		59
**Ki-67**		
≤20%		33
>20%		95
**Subtype**		
Luminal		48
Triple-negative		42
HER2-enriched		20
In situ		18
**Lymph node**		
0		70
>0		58
