# Supplementary material for: Circulating tRNA-derived small RNAs (tsRNAs) signature for the diagnosis and prognosis of breast cancer
Source: NPJ Breast Cancer. 2021 Jan 5;7:4. doi: 10.1038/s41523-020-00211-7 (PMC7785726; doi:10.1038/s41523-020-00211-7)
Supplement: Supplementary file 2 — Reporting Summary [file 41523_2020_211_MOESM2_ESM.pdf]

nature research

Corresponding author(s): NPJBCANCER-00713

Last updated by author(s): Jul 22, 2020

Reporting Summary

Nature Research wishes to improve the reproducibility of the work that we publish. This form provides structure for consistency and transparency in reporting. For further information on Nature Research policies, see Authors & Referees and the Editorial Policy Checklist.

Statistics

For all statistical analyses, confirm that the following items are present in the figure legend, table legend, main text, or Methods section.

n/a	Confirmed	
☐	☒	The exact sample size (n) for each experimental group/condition, given as a discrete number and unit of measurement
☐	☒	A statement on whether measurements were taken from distinct samples or whether the same sample was measured repeatedly
☐	☒	The statistical test(s) used AND whether they are one- or two-sided<br>*Only common tests should be described solely by name; describe more complex techniques in the Methods section.*
☐	☒	A description of all covariates tested
☐	☒	A description of any assumptions or corrections, such as tests of normality and adjustment for multiple comparisons
☐	☒	A full description of the statistical parameters including central tendency (e.g. means) or other basic estimates (e.g. regression coefficient) AND variation (e.g. standard deviation) or associated estimates of uncertainty (e.g. confidence intervals)
☐	☒	For null hypothesis testing, the test statistic (e.g. F, t, r) with confidence intervals, effect sizes, degrees of freedom and P value noted<br>*Give P values as exact values whenever suitable.*
☒	☐	For Bayesian analysis, information on the choice of priors and Markov chain Monte Carlo settings
☒	☐	For hierarchical and complex designs, identification of the appropriate level for tests and full reporting of outcomes
☒	☐	Estimates of effect sizes (e.g. Cohen's d, Pearson's r), indicating how they were calculated

Our web collection on statistics for biologists contains articles on many of the points above.

Software and code

Policy information about availability of computer code

Data collection	No software was used.
Data analysis	No software was used.

For manuscripts utilizing custom algorithms or software that are central to the research but not yet described in published literature, software must be made available to editors/reviewers. We strongly encourage code deposition in a community repository (e.g. GitHub). See the Nature Research guidelines for submitting code & software for further information.

Data

Policy information about availability of data

All manuscripts must include a data availability statement. This statement should provide the following information, where applicable:

- Accession codes, unique identifiers, or web links for publicly available datasets
- A list of figures that have associated raw data
- A description of any restrictions on data availability

All remaining data and materials during the study are available from the authors upon reasonable request.

Field-specific reporting

Please select the one below that is the best fit for your research. If you are not sure, read the appropriate sections before making your selection.

☒ Life sciences ☐ Behavioural & social sciences ☐ Ecological, evolutionary & environmental sciences

For a reference copy of the document with all sections, see nature.com/documents/nr-reporting-summary-flat.pdf

Life sciences study design

All studies must disclose on these points even when the disclosure is negative.

Sample size	All participants were randomly recruited from the First Affiliated Hospital with Nanjing Medical University between January and December 2015. A total of 120 patients with BC without neoadjuvant therapy and 112 healthy controls were enrolled in this study.
Data exclusions	We excluded patients who received neoadjuvant therapy.
Replication	All experiments were repeated at least three times to verify the reproducibility of the experimental findings.
Randomization	This is a retrospective population-based cohort study.
Blinding	This is a retrospective population-based cohort study.

Reporting for specific materials, systems and methods

We require information from authors about some types of materials, experimental systems and methods used in many studies. Here, indicate whether each material, system or method listed is relevant to your study. If you are not sure if a list item applies to your research, read the appropriate section before selecting a response.

Materials & experimental systems

n/a	Involved in the study	
☒	☐	Antibodies
☐	☒	Eukaryotic cell lines
☒	☐	Palaeontology
☒	☐	Animals and other organisms
☐	☒	Human research participants
☐	☒	Clinical data

Methods

n/a	Involved in the study	
☒	☐	ChIP-seq
☒	☐	Flow cytometry
☒	☐	MRI-based neuroimaging

Eukaryotic cell lines

Policy information about cell lines

Cell line source(s)	All cell lines were obtained from ATCC (Manassas, USA).
Authentication	All cell lines used were authenticated.
Mycoplasma contamination	All cell lines tested were negative for mycoplasma contamination.
Commonly misidentified lines (See ICLAC register)	None.

Human research participants

Policy information about studies involving human research participants

Population characteristics	A total of 120 patients with BC without neoadjuvant therapy and 112 healthy controls were enrolled in this study.
Recruitment	All participants were recruited randomly.
Ethics oversight	The First Affiliated Hospital with Nanjing Medical University.

Note that full information on the approval of the study protocol must also be provided in the manuscript.

Clinical data

Policy information about clinical studies

All manuscripts should comply with the ICMJE guidelines for publication of clinical research and a completed CONSORT checklist must be included with all submissions.

Clinical trial registration	This is a retrospective population-based cohort study.
Study protocol	This is a retrospective population-based cohort study.

Data collection	Data for all participants were collected from the First Affiliated Hospital with Nanjing Medical University between January and December 2015.
Outcomes	The primary outcome in the present study was the expression levels of identified tsRNAs in plasma.
